# ISSR Analysis of Genetic Diversity and Structure of Plum Varieties Cultivated in Southern China

**DOI:** 10.3390/biology8010002

**Published:** 2018-12-21

**Authors:** Weifeng Wu, Faxing Chen, Kaiwun Yeh, Jianjun Chen

**Affiliations:** 1 Institute of Subtropical Fruit, Fujian Agriculture and Forestry University, Fuzhou 350002, China; wuweifeng710@163.com (W.W.); ykwbppp@ntu.edu.tw (K.Y.); 2 College of Horticulture, Fujian Agriculture and Forestry University, Fuzhou 350002, China; 3 Institute of Plant Biology, National Taiwan University, Taipei 106, Taiwan; 4 Mid-Florida Research and Education Center, University of Florida, IFAS, Apopka, FL 32703, USA

**Keywords:** Chinese plums, genetic diversity, genetic structure, *Prunus salicina*, simple-sequence repeat markers (ISSR), plum

## Abstract

Plums (*Prunus* spp.) are important deciduous fruit crops in the world. China is a major producer of *P. salicina* Lindl., but the genetic relationship of Chinese plums in key production regions remain unclear. In this study, 14 University of British Columbia (UBC) inter simple sequence repeats (ISSR) primers were used to analyze 33 plum varieties cultivated in Fujian Province to determine their genetic diversity and population structure. A total of 146 bands were generated, of which 130 were polymorphic. Mean percentage of polymorphic bands was 89.04%, Shannon’s information index value was 0.38, and the Nei’s genetic index value was 0.24. Using unrooted trees (Neighbor-Joining method), 33 varieties were classified into four groups. Split graph separated them into two major groups, each with two subgroups. The two phylogenetic trees indicate that environmental or natural selection pressure is an important factor influencing their genetic relationship. Analysis of population structure revealed that they have frequent genetic exchanges among closed subpopulations; thus, genetic variation mainly occurs within the population. Additionally, based on the phylogenetic analysis and unique morphological characteristics of fruits, we propose that the Chinese landrace Nai could contribute significantly to development of the famous variety Wickson.

## 1. Introduction

Plums are deciduous fruit crops grown ubiquitously in China, America, Japan, and European countries [1]. Most commercially produced plums are either Chinese plum (*Prunus salicina* Lindl, 2n = 2x = 16) or European plum (*Prunus domestica* Lindl, 2n = 6x = 48) [2]. Chinese plum originates from the Yangtze River Basin in China and was introduced to Japan; it also called Japanese plum because the first import of this crop to the United States (U.S.) in 1870 was from Japan. Luther Burbank used *P. salicina* to hybridize with other species, including *P. americana*, *P. angustifolia*, *P. munsoniana*, and *P. simonii*, resulting in the development of more than 100 varieties [3]. Modern Chinese plum varieties are largely *P. salicina*, but some may have genetic makeup of other species due to the introduction of Luther Burbank’s varieties [4]. The origin of *P. domestica* remains unclear, but it is accepted that *P. spinosa* and *P. cerasifera* are involved in the genesis of European plum [2]. Through open pollination and selection as well as breeding, a large number of varieties of *P. domestica* were released [3].

Fruits of *P. salicina* are almost exclusively used for fresh consumption, whereas fruits of *P. domestica* are generally used for processing. Plums are used to make jam, juice liquor, and brandy. Concentrated juices of plum are also used for medicinal purposes as a laxative. Plum breeding has resulting in the release of yellow, red, purple, and black plums, and they are important sources of flavonoids, anthocyanins, carotenes, and polyphenolic acids [5]. Some blood-colored Chinese plums contain anthocyanins up to 300 mg per 100 g, exceeding or comparable to those of many berry fruits [4]. Due to their nutritional value, the production of plums increased greatly over the years [6]. The Food and Agriculture Organization Corporate Statistical Database showed that the total world production of plum and sloe in 2016 was 12 million metric tonnes, of which China produced 55% [7].

**Figure 1:**
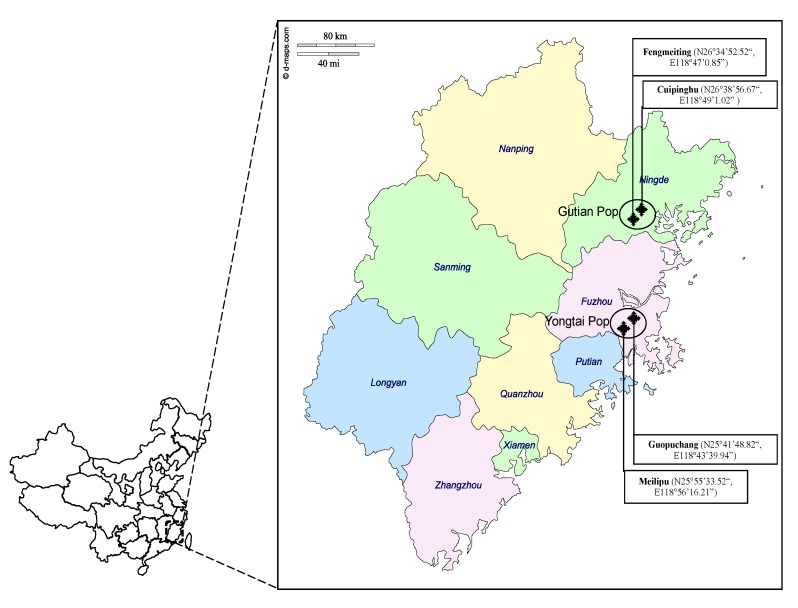
Geographical regions of 33 plum varieties (Table 1) collected from Gutian as Gutian population (Cuipinghu subpopulation and Fengmeiting subpopulation) and Yongtai as Yongtai population (Guopuchang subpopulation and Meilipu subpopulation) in Fujian Province, China.

China has rich plum germplasm resources. Fujian Province, located on the southeast coast of China (Figure 1), has more than 700 years of production history and remains a major plum production region in China [8,9,10]. Traditionally, there are two major types of Chinese plum: Naili and Furongli [11]. Naili includes Hua Nai and Qing Nai [12]. In addition to the local varieties or landraces, many varieties have been introduced from the U.S. and Japan since the early 1900s. The introduction brought about duplicated and mislabeled accessions [13]. Additionally, production of local varieties along with introduced varieties have resulted in the decline of landraces. For example, the Chinese Nai, which has been cultivated for more than nine hundred years in Southeast China, has fruit with a small pit, large heart-shape, and yellowish red skin, indicating that its fruit phenotype is similar to that of Wickson plum. Wickson was bred by Burbank through a cross between Burbank (*P. salicina*) and Simon (*P. Simonii*) or Kelsey [14]. Furthermore, the introduction might also lead to the selection of new varieties from open-pollinated populations. Therefore, there is critical need for establishing efficient methods for distinguishing the current varieties and their genetic relationships.

Molecular marker techniques are effective tools for analyzing genetic diversity and relationships of plants [15,16]. Some *P. domestica* genetic resources were assessed by random amplified polymorphic DNA (RAPD) [17,18,19] and microsatellite markers [20]. Selected genetic resources of *P. salicina* were analyzed by RAPD [21,22], amplified fragment length polymorphisms (AFLP) [23], and inter-simple sequence repeats (ISSR) [13,23,24,25]. Among the molecular markers, ISSR generally shows higher reproducibility and higher percentage of polymorphism than the other markers [23,26]. Plum genetic resources that have been analyzed are those primarily collected from European countries, Japan, and USA. Thus far, there is only one report on analysis of Chinese plum germplasm [13]. Using ISSR markers, the authors studied the genetic diversity of 104 plums and related accessions from the Chinese National Germplasm Repository for Plums and Apricots located in Xiongyue, Liaoning Province. Those genetic resources are predominately from Northern China [13]. 

Fujian has over 700 years of history in plum cultivation and is still a major plum production province in China. Plum varieties or genetic resources from Fujian may represent Southern Chinese plum genetic resources. We believe that these genetic resources could significantly differ from those of Northern China. However, varieties cultivated in Southern China have not been well studied. The objective of this study was to use ISSR to analyze the genetic diversity and structure of plum varieties cultivated in Southern China, primarily Fujian. A better understanding of their genetic relationships could improve plum germplasm conservation and utilization and lead to new cultivar development. 

## 2. Materials and Methods

### 2.1. Materials and DNA Extraction

A total of 33 varieties were collected from two regions of Fujian province (Table 1, Figure 1). One is Gutian, located at the northeastern part of Fujian province, including Cuipinghu and Fengmeiting areas. Ten varieties were collected from the former and 12 from the latter area. The other region is Yongtai, located in the eastern part of Fujian province, including Meilipu and Guopuchang areas where three and eight varieties were collected, respectively. The two regions are separated by Fuzhou city, mountains and valleys and about 175 km apart. Collected young leaves were frozen in liquid N and stored at −80 ℃ for DNA extraction. 

DNA was extracted following the protocol of Liu [13] with modifications. Briefly, 0.1 g of frozen leaves was put into mortar and ground to a fine powder in liquid N. Powder was transferred into a 1.5 mL of tube with addition of 650 µL of extraction buffer (2% CTAB, 1.4 M NaCl, 100 mM Tris-HCl, 20 mM EDTA, 2% ß-ME, and 1% PVP) and incubated at 65 ℃ for 30 min. DNA was extracted with 650 µL of chloroform/isoamyl alcohol (24:1, *v*/*v*). The DNA suspension was mixed gently and centrifuged at 13,000× *g* (4 ℃) for 15 min. The top aqueous phase was collected into a new tube with addition of 2 µL of RNase (10 mg/mL), mixed, and incubated at 37 ℃ for 30 min. DNA was extracted once more with one volume of the chloroform/isoamyl mixture, the top aqueous phase was collected again, and DNA was precipitated with two volumes of cold ethanol. Resultant DNA pellet was washed three times with 70% ethanol and dissolved in 50 µL of sterile water after complete evaporation of ethanol. DNA quality was checked on a 1% agarose gel, and the concentration was determined by NanoDrop Lite Spectrophotometer with Printer (Thermo Fisher Scientific, Waltham, MA, USA). A portion of the DNA was diluted to 20 ng/µL for use; both the stock and diluted portions were stored at −40 ℃. 

### 2.2. Primer Screening and PCR Amplification

A total of 100 ISSR primers were initially screened, of which 14 University of British Columbia (UBC) primers that produced clear and highly polymorphic bands were selected (Table 2). PCR reaction mixtures had a total volume of 20 μL containing 60 ng of DNA template, 0.25 μL primer, and 10 μL SuperMix. All reactions were conducted in T100TM Thermal Cycler (Bio-Rad, Hercules, CA, USA) with the following conditions: 5 min at 94 ℃ for initial denaturation, 40 cycles of 45 s denaturation at 94 ℃, 1 min annealing at the primer specific temperature (Table 2), and 2 min extension at 72 ℃, followed by a final extension for 5 min at 72 ℃. Each reaction was repeated twice. Amplified products were separated by electrophoresis on 2% agarose gel (Biowest, Madrid, Spain) in 1× TAE buffer under a voltage of 130 V/cm for ≈35 min. The gels were photographed using the BIO-RAD GelDocXR + Imaging system (Bio-Rad, Hercules, CA, USA).

### 2.3. Analysis of Genetic Diversity

Amplified reproducible bands were scored as present (1) or absent (0) to create a binary matrix. To analyze genetic diversity of the varieties, the percentage of polymorphic bands (PPB), the observed number of alleles (Na) and effective number of alleles (Ne), Nei’s gene diversity index (h), Shannon’s Information index (I) were calculated using POPGENE v.1.32 [27]. Gene flow (Nm) and Nei’s Analysis of Gene Diversity in subdivided populations were analyzed using GenAlexv.6.0 [28]. An unrooted tree based on neighbor-joining (NJ) and a split network phylogenetic tree (NNet) using Splitstreev.4.6 [29] were constructed. The distribution of incompatible splits based on uncorrected *p* distance were inferred, which provided a split graph through NNet analysis. Nei’s analysis of genes in subdivided populations via POPGENE v.1.32 [27] was also used to calculate total genotypes diversity (Ht), within population diversity (Hs), mean coefficient of gene differentiation (Gst) value, and genetic differentiation coefficient (Φst) within a region and among regions. 

### 2.4. Analysis of Genetic Structure

The Structure version 2.2.3 [30], which is based on a Bayesian model, was used to infer population structure and assign varieties to specific populations based on ISSR genotypes. This approach is suitable for analysis of diploids and polyploids. We estimated *K*, the number of reconstructed panmictic populations (RPPs) of individuals, by testing *K* (log-likelihood) = 2–7 for all varieties, assuming that they were from unknown origin. Ten independent runs were conducted for each *K*. A burn-in period of 200,000 iterations was applied. Subsequently, structure harvester (http://taylor0.biology.ucla.edu/structureHarvester) [31], which implements the Evanno method [32], was used to estimate the most probable *K* value and Q value (representing that percent value of assigning genotypes to a specific population), the run with the maximum likelihood was used to assign genotypes to specific clusters [33]. 

## 3. Results 

### 3.1. ISSR Polymorphism and Genetic Diversity

The 14 ISSR primers generated a total of 146 scorable bands, ranging from 5 to 15 per primer (Table 2). An example of ISSR profiles of 33 varieties produced by UBC811 is presented in Figure S1. Statistical analysis showed that there were 130 polymorphic bands and 16 monomorphic bands. Genetic diversity of *P. salicina* across 33 varieties was high (PPB = 89.04%). The observed and effective numbers of alleles (Na and Ne) were 1.95 and 1.39, respectively. The Nei’s genetic index value (h) was 0.24, and the Shannon’s information index value (I) was 0.38. The genetic diversity of varieties collected from Gutian region (PPB = 86.30%, Na = 1.86, Ne = 1.38, h = 0.23, and I = 0.36) was higher than those collected from Yongtai region (PPB = 71.23%, Na = 1.71, Ne = 1.33, h = 0.20, and I = 0.32) (Table 3).

### 3.2. Genetic Relationship and Differentiation

**Figure 2:**
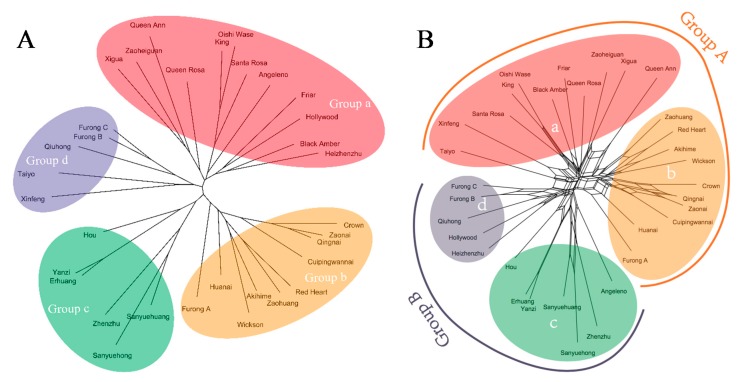
Phylogenetic trees of 33 plum varieties constructed based on ISSR data: An unrooted tree established using the NJ method (**A**) and a tree constructed using the NNet method calculated from the uncorrected *p* distances (**B**).

An unrooted tree based on NJ method was constructed for 33 varieties (Figure 2A). The tree showed that the varieties branched into four groups (a, b, c, and d), which largely coincided with the four regions: Cuipinghu, Fengmeiting, Meilipu, and Guopuchang, where the varieties were collected, with a few exceptions. There were 12 varieties in group a, which were collected from Cuipinghu with exception of Angeleno, Hollywood, and Heizhenzhu that were collected from Meilipu. Varieties Angeleno, Black Amber, Friar, Hollywood, King, Oishi Wase, Queen Ann, Queen Rosa, and Santa Rosa, which were introduced from the U.S. or Japan (Table 1), were clustered in the group a (Figure 2A). Group b consisted of 10 varieties that were most collected from Fengmeiting. This group contained a mixture of varieties from Japan, US, and Fujian. There were six varieties in group c: two collected from Fengmeiting, two from Cuipinghu, and the other two from Guopuchang, they all were Chinese varieties, native to either Fujian, Guangdong or Taiwan. Five varieties were in group d, which were collected from Guopuchang, they were Chinese varieties, except Taiyo, which was native to Japan. Additionally, fruit color and size varied among varieties (Table 1), which appeared to have no relationships with the classification derived from NJ method (Table 1). 

The NNet analysis based on split networks provides additional topology-related parameters to phylogenetic analysis. The NNet analysis divided the 33 varieties into two groups, and each group was divided into two subgroups. Group A was dominated by Gutian population, including subgroups a and b, while group B was dominated by Yongtai population, consisting of subgroups c and d. Results of NNet analysis were largely similar to those of NJ analysis (Figure 2A vs. Figure 2B) except for five varieties: Angeleno, Heizhenzhu, Hollywood, Taiyo, and Xinfeng that were grouped differently. Nei’s analysis of gene in subdivided populations using POPGENE v.1.32 showed that the value for total 33 genotypes diversity (Ht) was 0.239, while within population diversity (Hs) was 0.219. Mean coefficient of gene differentiation (Gst) value (0.0835) indicated that 91.65% of the genetic diversity existed in the regional population (Table 4). The analysis of molecular variance (AMOVA) showed that the genetic differentiation coefficients (Φst) within and among the region were 88% and 12% (*p* = 0.001), respectively (Table S1 and Table 4). This result was similar to that of Nei’s genetic diversity. Additionally, the gene flow index (Nm) among regions was up to 5.488 (Table 4). Furthermore, analysis of the varieties based on their origins (China, Japan, and the U.S.) also showed that the genetic differentiation coefficient (Φst) within population was 90% compared to 10% among the populations (*p* = 0.001) (Table S2).

### 3.3. Genetic Structure

**Figure 3:**
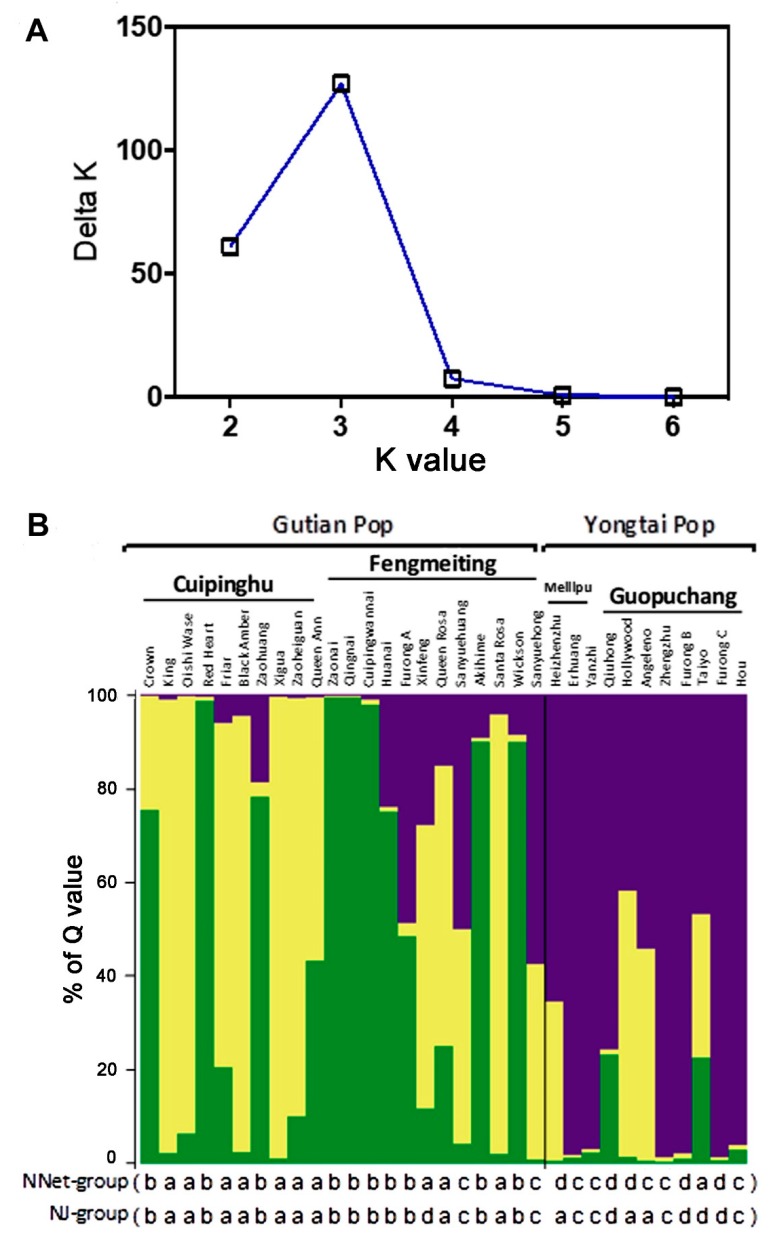
The Bayesian analysis of genetic structure of 33 varieties. (**A**) Plot from Structure Harvester showed *K* = 3 inferred by the estimation of the Δ*K* parameter. (**B**) Bar plot of the results from the Bayesian analysis (*K* = 3) of 33 varieties. Each variety is represented by a vertical bar, which is partitioned into colored segments whose length depends on the estimated membership fraction (Q) in each population. Varieties are assigned to a specific population when the highest Q is higher than 0.6. Three populations are colored in yellow as the Cuipinghu population, green as the Fengmeiting population, and purple as the Guopuchang and Meilipu population. Varieties classified into a, b, c, and d groups by NNet and NJ methods (Figure 2) are listed under the bar plot.

Bayesian analysis assigned individuals to RPPs based on genotype, and the Structure version 2.2.3 program [30] estimated the most likely number of cluster (*K*) by calculating the log probability of data for each value of *K* and using Δ*K* statistics described by Evanno et al. [32]. Results showed there was a sharp peak Δ*K* at *K* = 3 (Figure 3A), which indicated there were three genetic components, which provided the rationality for classifying the 33 genotypes into three RPPs or populations. In the bar plot of Figure 3B, varieties colored by yellow mainly belonged to the Cuipinghu population, by green largely as the Fengmeiting population, and purple as the Guopuchang and Meilipu populations. Comparing the results of the Structure analysis with NJ and NNet analyses (Figure 3B), varieties clustered in group a in NNet or NJ analysis were primarily yellow in color in the bar plot, group b in green, and groups c and d in purple. The three genetic components were based on the probability of germplasm originated from a specific population. Twenty-five genotypes were relative single according to the ‘Q > 0.6 relative to that of the single’ standard [37,38], and another eight genotypes were more complex, which comprise of Queen Ann, Furong A, Queen Rosa, Sanyuehuang, Sanyuehong, Hollywood, Angeleno, and Taiyo.

## 4. Discussion

It has been well documented that ISSR is a cost-effective technique for detection of DNA polymorphisms and for identification and conservation of plum genetic resources [13,23,25]. The percentage of polymorphic bands (PPB), Nei’s gene diversity index (h), and Shannon’s information index (I) are key parameters to measure the genetic diversity. Our study shows that the 33 plum varieties are genetically diverse as the values of PPB, h, and I are equal to 89.04%, 0.2416, and 0.3794, respectively. These values are higher than those reported by Carrasco et al. [25] (PPB = 88.4%, h = 0.15, I = 0.27) which were assessed using ISSR and greater than those of 26 genotypes in Greece (PPB = 77.33%) analyzed using both ISSR and RAPD [39]. An explanation for the higher genetic diversity could be due to the introduction of varieties from Japan and U.S. in particular where many varieties were developed through interspecific hybridization between *P. salicina* and other species, such as *P. americana*, *P. angustifolia*, *P. munsoniana*, and *P. simonii* [3]. Fujian is located on the coast of southeast China, which is one of first geographical areas for introducing new plants and new varieties and is also an important region for plum production. It is unknown when some varieties developed from Japan and the U.S. were introduced to Fujian. Wickson and Santa Rosa were released in 1892 and 1906, respectively. Angeleno and Friar were developed in the 1960s; Queen Rosa and Black Amber became available in the 1970s and 1980s, respectively (Table 1). It is reasonable to assume that these varieties might have been introduced to Fujian for near or more than a half century. Their introduction, along with their cultivation with local varieties and their use for plum breeding, could significantly increase the genetic diversity of the current plum genetic resources in Fujian. 

Genetic relationships of the 33 varieties are presented in Figure 1. The unrooted tree based on NJ method divided them into four groups (Figure 1A), many of which are coincided with geographic locations. Unrooted phylogenetic trees based on genetic distances of genotypes are generally used for inferring evolutionary relationships. Most of such phylogenetic trees are unable to display some parameters, such as horizontal gene transfer, hybridization, and recombination [29]. As a result, phylogenetic networks (NNet) analysis was used to explain visualized conflicting signals in the data sets, whether they arise from sampling error or genuine recombination [40,41]. The NNet analysis categorized the cultivars into two groups, which again show the territoriality (Figure 1B). Group A was dominated by Gutian population comprising Fengmeiting and Cuipinghu subgroups. Group B was dominated by Yongtai population consisting of Meilipu and Guopuchang subgroups. There are some disagreements between unrooted phylogenetic tree and NNet methods in clustering a few varieties. The unrooted tree shows that Sanyuehong, Sanyuehuang, and Xinfeng derived from Gutian were in the Yongtai population clusters, while Angeleno and Hollywood from Yongtai were situated in the Gutian population clusters. The NNet analysis shows that Heizhenzhu, Sanyuehong, and Sanyuehuang from Gutian were classified into the Yongtai population clusters. As the NNet analysis based on split networks adds extra topology-related parameters to phylogenetic analysis, allowing the constructed network to fit the data better than unrooted trees [29,40], the results of phylogenetic networks are inclined to territoriality than that of the NJ phylogenetic tree. Group A (Figure 1B) has 21 varieties, of which 11 are introduced from either Japan or the U.S. and the rest are native to Fujian, except Zaohuang, which is from Anhui Province. Group B has 12 varieties, and they are largely native to Fujian or South China, except Angeleno and Hollywood, which were introduced from the U.S. 

In general, genetically close individuals are often clustered together [42]. This is also true in our analysis. For example, Red Heart, selected from a cross between Duarte and Wickson, is grouped with Wickson; Black Amber (Friar x Queen Rosa) is clustered with Friar and Queen Rosa; and Queen Rosa (Queen Ann x Santa Rosa) is situated with Queen Ann and Santa Rosa in the same cluster (Figure 1). However, Furongli is a landrace of Fujian, Furong A (large-type) is not clustered together with Furong B and Furong C. A possible reason could be due to geographic barrier resultant genetic variation. Fujian is a mountainous province and many mountains are higher than 1000 m above sea level. Within the same region, the continuous and natural selection pressure could be an important factor for genetic adaptation. The association between genotypic differentiation and environmental factors within plant populations was also observed through morphological, isozyme, and molecular markers [43,44,45,46]. 

A high level of gene flow limited genotypic differentiation within populations. In contrast, a lower level of gene flow induced the adaptability of populations on the small ecological environments to promote genetic isolation within populations [47]. The results of POPGENE analysis (Gst = 0.0835) and AMOVA (Φst, within population = 88%) (Table S1) show that the genetic variation of 33 genotypes mainly occurred within the population. Further analysis based on three origins (China, Japan, and the U.S.) also shows that the genetic variation primarily occurs within population (Table S2). Similar results were reported by other authors [25,42,48]. The highly genetic variation within the population could be attributed to several factors: (1) The pollination biology of plums as they are unable to bear fruit parthenocarpically and require cross-pollination due to high self-incompatibility. On the other hand, varieties differ in chilling requirements for flowering, pollen is transferred by insects, and the best time for pollination is only 1–3 days after the opening of flower [49]. These factors can significantly affect pollination. (2) Nonrandom mating pattern occur among varieties. Some require pollen from specific varieties. For example, Queen Ann prefers pollen from Santa Rosa, and Queen Rosa requires pollen from Friar, Santa Rosa, and Wickson [49]. (3) The introduced varieties from Japan and the U.S., their production along with local varieties, and their use in breeding increased genetic variation within geographic locations. (4) Potential genetic drift due to small population size, such as those in group B and also mountainous barriers. All these factors and their interactions could increase genetic variation within the population. The gene flow index Nm was high (Nm = 5.488). This is probably due to the introduction of genetic resources, such as those from Japan and the U.S., and their use in selection and breeding of plum varieties in Fujian. 

The population genetic structures of the 33 varieties were evaluated by Structure 2.2.3. The results indicated that the varieties had three genetic components based on the probability of germplasm originated from a specific population. Thus, there is discrepancy between the results of structure (optimal *K* = 3) and clustering (2 or 4 main groups). A possible explanation about the difference could be due to the low resolution of cluster analysis. Further study is needed to clarify the difference. Nevertheless, 26 varieties were relatively single or genetically pure according the ‘Q > 0.6 relative to that of the single’ standard [37,38], suggesting they might have limited genetic exchanges with other varieties. The other eight varieties: Queen Ann, Furong A, Queen Rosa, Sanyuehuang, Sanyuehong, Hollywood, Angeleno, and Taiyo had Q < 0.6, which may indicate that they are genetically admixed or other species could contribute to their origins. The composition of Gutian population was relatively more complex than that of the Yongtai population at the population level. This could be attributed to the fact that the Gutian population is composed of more introduced varieties from Japan and the U.S., or their incorporation into breeding or natural selection, resulting in increased heterozygosity. 

Another note is that Nai used to be considered a hybrid derived from peach and plum based on its inner plum’s quality and peach’s outer form. After isoenzyme and RAPD molecular analyses, Nai was reconsidered to be a local variety or landrace of *P. salicina* [50,51]. In the present study, there were five Nai varieties—Crown, Cuipingwannai, Huanai, Qingnai, and Zaonai—which were clustered with other plum varieties. Our result provides additional evidence supporting that Nai belongs to *P. salicina.* Furthermore, these Nai varieties are closely associated with Red Heart (Duarte x Wickson) and Wickson (Burbank x Kelsey) in both phylogenetic trees (Figure 1). Based on Karp [14], Kelsey was imported from Japan to the U.S. in the late 1800s [52]. It produces large and heart-shaped fruit with skin ranging from green to yellow or red. Subsequently, over 100 plum cultivars were released by Burbank, including Wickson. Wickson has large, hear-shaped fruit with a small pit, yellow or red skin, which is quite similar to those of Nai. As documented by Matsumoto (1978) [53], *P. salicina* was brought to Japan as a gift by Buddhism 2000 years ago. Based on the phylogenetic analysis and their morphology, we propose that Chinese plum Nai contribute greatly to development of Wickson. 

## 5. Conclusions

This study used 14 UBC ISSR primers to analyze genetic diversity and structure of 33 varieties of *Prunus salicina* cultivated in Fujian Province, China. The 14 primers generated a total of 146 bands, of which 130 were polymorphic. Results showed that the mean percentage of polymorphic bands was 89.04%, Shannon’s information index value was 0.38, and the Nei’s genetic index value was 0.24, suggesting the 33 varieties are genetically diverse. Based on the NJ method, 33 varieties were categorized into four groups, whereas NNet analysis classified them into two major groups, each with two subgroups. Both classifications suggest the territoriality, or the location of their cultivation significantly influenced their genetic relationships. Population structure analysis indicated that frequent genetic exchanges occurred among closed subpopulations. As a result, genetic variation mainly occurs within the population. Additionally, based on unique morphological characteristics of fruits and phylogenetic analysis, we postulate that the Chinese landrace Nai could be the ancestor of the famous variety Wickson developed by Luther Burbank in 1892.

## Figures and Tables

**Table 1:** Sources of ISSR analyzed *Prunus salicina* varieties cultivated in Fujian province, China.

No.	Cultivar	Background	Origin/Originator ^z^	Loc ^y^	Key Traits
1	Crown	A mutant of *P. salicina*	Gutian, Fujian, China	Ⅰ^1^	Large fruit, yellowish skin and flesh, mature May–June, high yield
2	King	*P. salicina*	Japan	Ⅰ^1^	Dark red skin, orange-colored flesh, sweet taste, mature mid-June
3	Oishi Wase	*P. salicina*	Japan	Ⅰ^1^	Small fruit, green-yellowish red skin, red flesh, mature early July, cold and drought tolerance
4	Red Heart	Duarte Wickson	USA	Ⅰ^1^	Large fruit, reddish skin and flesh, mature mid-July, drought and cold tolerance
5	Friar	Gaviota × Nubiana	Fresno, CA, USA	Ⅰ^1^	Large fruit, black skin, white flesh, mature early September, cold tolerance, released 1968
6	Black Amber	Fiar × Queen Rosa	Fresno, CA, USA	Ⅰ^1^	Dark, purple blue skin, yellow flesh, mature early August, cold tolerance, released 1980
7	Zaohuang	*P. salicina*	Anhui, China	Ⅰ^1^	Small fruit, yellowish skin and flesh, mature mid-July, cold tolerance
8	Xigua	*P. salicina*	Fujian, China	Ⅰ^1^	Small, purple skin and flesh, mature May, high yield
9	Zaoheiguan	*P. salicina*	Fujian, China	Ⅰ^1^	Large fruit, black skin, red flesh, mature June
10	Queen Ann	Gaviota × Eldorado	CA, USA	Ⅰ^1^	Large fruit, black skin, light yellowish flesh, mature July–August
11	Zaonai	*P. salicina*	Fujian, China	Ⅰ^2^	Large fruit, light green skin, yellow flesh, mature July
12	Qingnai	*P. salicina*	Fujian, China	Ⅰ^2^	Large fruit, green-yellowish skin, light-yellow flesh, mature late July
13	Cuipingwannai	*P. salicina*	Gutian, Fujian, China	Ⅰ^2^	Large fruit, green skin, yellow flesh, mature August
14	Huanai	*P. salicina*	Sha County, Fujian, China	Ⅰ^2^	Mid-sized fruit, green-yellowish to red skin, reddish flesh, mature June–July
15	Furong A	*P. salicina*	Fujian, China	Ⅰ^2^	Large fruit, red skin, dark red flesh, mature mid-July
16	Xinfeng	*P. salicina*	Fujian, China	Ⅰ^2^	Mid-sized, purple skin, yellowish flesh, mature June
17	Queen Rosa	Queen Ann × Santa Rosa	Fresno, CA, USA	Ⅰ^2^	Large fruit, purple skin, light-yellowish flesh, mature August, drought and cold tolerance, released 1972
18	Sangyuehuang	*P. salicina*	Guangdong, China	Ⅰ^2^	Small fruit, yellowish skin and flesh, mature late March and April
19	Akihime	*P. salicina*	Japan	Ⅰ^2^	Large fruit, purple skin, yellow flesh, mature mid-September
20	Santa Rosa	*P. salicina*	Burbank, USA	Ⅰ^2^	Red-skin fruit with gold flesh, released 1906
21	Wickson	*Burbank* × Kelsey	Burbank, USA	Ⅰ^2^	Large, light yellowish skin and flesh, mature August, released 1892
22	Sanyuehong	*P. salicina*	Guangdong, China	Ⅰ^2^	Small fruit, red skin, red flesh, mature April
23	Heizhenzhu	*P. salicina*	Taiwan, China	Ⅱ^1^	Large fruit, black skin, red flesh, mature mid-August
24	Erhuang	*P. salicina*	Fujian, China	Ⅱ^1^	Mid-sized fruit, yellow skin and flesh, mature July
25	Yanzhi	*P. salicina*	Fujian, China	Ⅱ^1^	Small fruit, purplish skin and flesh, mature June
26	Qiuhong	*P. salicina*	Shandong, China	Ⅱ^2^	Mid-sized fruit, purplish-red skin, green-yellowish flesh, mature mid-August
27	Hollywood	*P. salicina*	USA	Ⅱ^2^	Mid-sized fruit, purplish red skin, red flesh, mature July, high yield
28	Angeleno	Op of Queen Ann ^x^	Garabadien, CA, USA	Ⅱ^2^	Large fruit, red skin, light yellow flesh, mature late-September, cold tolerance, released 1967
29	Zhenzhu	*P. salicina*	Taiwan, China	Ⅱ^2^	Small fruit, purplish skin, yellow flesh, mature August
30	Furong B	*P. salicina*	Fujian, China	Ⅱ^2^	Mid-sized fruit, red skin, dark red flesh, mature mid-July
31	Taiyo	*P. salicina*	Japan	Ⅱ^2^	Mid-sized purple fruit, mature August
32	Furong C	*P. salicina*	Fujian, China	Ⅱ^2^	Mid-sized fruit, red skin, dark red flesh, mature August
33	Hou	*P. salicina*	Yongtai, Fujian, China	Ⅱ^2^	Mid-sized fruit, yellow to reddish skin, red flesh, mature late June

^z^ Origin refers to where the variety came from and originator means who developed the variety. ^y^ Loc. = Location: Gutian population as I (Ⅰ^1^: Cuipinghu subpopulation; Ⅰ^2^: Fengmeiting subpopulation), and Yongtai population as II (Ⅱ^1^: Meilipu subpopulation; Ⅱ^2^: Guopuchang subpopulation). ^x^ Selected from open-pollinated Queen Ann progenies.

**Table 2:** Total number of bands, number and percentage of polymorphic bands generated by 14 ISSR primers from 33 plum varieties.

Primer Code	Sequence	Annealing Temperature (°)	Total No. of Bands	No. of Polymorphic Bands	% of Polymorphic Bands (PPB)
UBC807	AGA GAG AGA GAG AGA GT	52	14	14	100.00
UBC810	GAG AGA GAG AGA GAG AT	52	14	14	100.00
UBC811	GAG AGA GAG AGA GAG AC	52	16	15	93.75
UBC815	CTC TCT CTC TCT CTC TG	54	10	9	90.00
UBC827	ACA CAC ACA CAC ACA CG	54	6	5	83.33
UBC835	AGA GAG AGA GAG AGA GYC	56	7	6	85.71
UBC840	GAG AGA GAG AGA GAG AYT	53	9	8	88.89
UBC841	GAG AGA GAG AGA GAG AYC	56	8	8	100.00
UBC842	GAG AGA GAG AGA GAG AYG	56	10	8	80.00
UBC843	CTC TCT CTC TCT CTC TRA	53	12	12	100.00
UBC855	ACA CAC ACA CAC ACA CYT	53	8	8	100.00
UBC873	GAC AGA CAG ACA GAC A	51	11	11	100.00
UBC880	GGA GAG AGG AGA	53	14	14	100.00
UBC888	BDB CAC ACA CAC ACA CA	53	7	7	100.00
Total			146	130	89.04

**Table 3:** Genetic variability among 33 plum varieties analyzed using 14 ISSR primers.

Pop ^z^	Na ^y^	Ne ^x^	H ^w^	I ^v^	No. of Polymorphic Loci	% of Polymorphic Bands
Gutian	1.8630	1.3842	0.2332	0.3615	126	86.30
Yongtai	1.7123	1.3348	0.2049	0.3188	104	71.23
All Pop	1.9521	1.3923	0.2416	0.3794	130	89.04

^z^ Pop = Population; ^y^ Na = Observed number of alleles; ^x^ Ne = Effective number of alleles [34]; ^w^ h = Nei’s gene diversity [35]; ^v^ I = Shannon’s Information index [36].

**Table 4:** Analysis of genetic structure in subdivided populations.

Index		Analytical Method
Ht ^z^	0.239	
Hs ^y^	0.219	Nei’s analysis of gene in subdivided populations
Gst ^x^	0.0835	
Nm ^w^	5.488	
Φst ^v^ (among Pops)	12%	The Analysis of Molecular Variance (AMOVA)
Φst (within Pops)	88%	

^z^ Ht = Total genotypes diversity; ^y^ Hs = Within population diversity; ^x^ Gst = Mean coefficient of gene differentiation value; ^w^ Nm = estimate of gene flow from Gst. Nm = 0.5 (1 − Gst)/Gst; ^v^ Φst = Genetic differentiation coefficient.

## Supplementary Materials

The following are available online at http://www.mdpi.com/2079-7737/8/1/2/s1, Figure S1: ISSR profiles of 33 plum varieties produced by the UBC811 primer, Table S1: Analysis of molecular variance (AMOVA) of 33 plum varieties based on two regions (Gutian and Yongtai), Table S2: Analysis of molecular variance (AMOVA) of 33 plum varieties based on their origins (China, Japan, and the U.S).
