# Supplementary material for: ISSR Analysis of Genetic Diversity and Structure of Plum Varieties Cultivated in Southern China
Source: Biology (Basel). 2018 Dec 21;8(1):2. doi: 10.3390/biology8010002 (PMC6466324; doi:10.3390/biology8010002)
Supplement: Supplementary file 1 [file biology-08-00002-s001.pdf]

Genetic Diversity and Structure of Plum Germplasms in Southern China Revealed by ISSR Markers

Weifeng Wu, Faxing Chen, Kaiwun Yeh and Jianjun Chen

Supplementary Figure and Tables

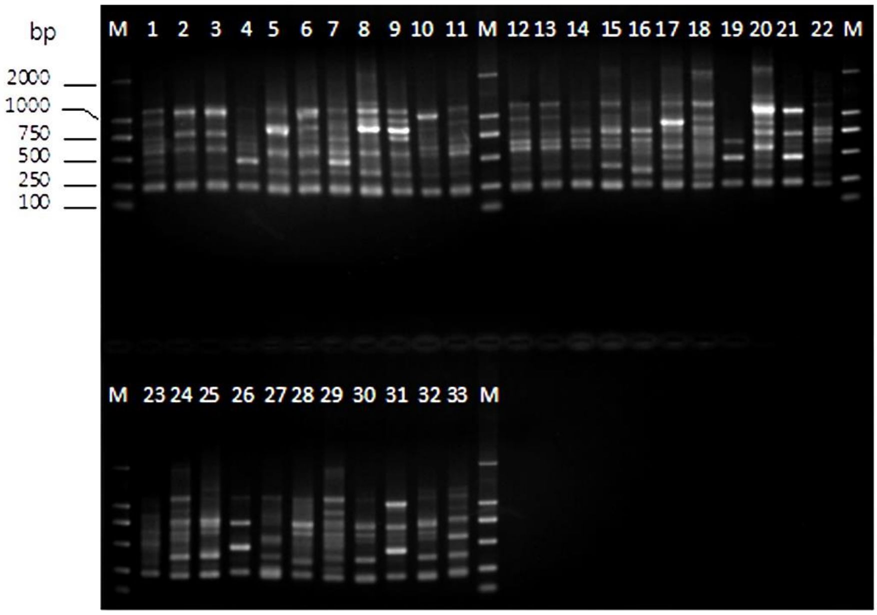

Figure S1. ISSR profiles of 33 plum varieties produced by the UBC811 primer. Lane M is marker and lanes 1–33 represent 1–33 varieties listed in Table 1.

Table S1. Analysis of molecular variance (AMOVA) of 33 plum varieties based on two regions (Gutian and Yongtai). Statistics include sums of squared deviation (SSD), mean squared deviation (MSD), estimated variance (Est. Var.), genetic differentiation coefficients (Φst), and the probability (*P*) of obtaining a more extreme component (Gutian and Yongtai).

Source	df	SSD	MSD	Est. Var.	Φst	*P*-value
Among Population	1	59.333	59.332	2.683	12%	0.001
Within Population	31	619.455	19.982	19.982	88%	
Total	32	678.788		22.665	100%	

Table S2. Analysis of molecular variance (AMOVA) of 33 plum varieties based on their origins (China, Japan, and the U.S). Statistics include sums of squared deviation (SSD), mean squared deviation (MSD), estimated variance (Est. Var.), genetic differentiation coefficients (Φst), and the probability (*P*) of obtaining a more extreme component.

Source	df	SSD	MSD	Est. Var.	Φst	*P*-value
Among Population	2	79.410	39.705	2.199	10%	0.001
Within Population	30	599.378	19.979	19.979	90%	
Total	32	678.788		22.178	100%	
